# Pictorial review of normal postoperative cross-sectional imaging findings and infectious complications following laparoscopic appendectomy

**DOI:** 10.1007/s13244-014-0369-6

**Published:** 2014-11-28

**Authors:** Massimo Tonolini, Federica Villa, Sonia Ippolito, Marina Petullà, Roberto Bianco

**Affiliations:** Department of Radiology, “Luigi Sacco” University Hospital, Via G.B. Grassi 74, 20157 Milan, Italy

**Keywords:** Appendectomy, Laparoscopic surgery, Acute appendicitis, Infectious complications, Abscess, Computed tomography (CT), Magnetic resonance imaging (MRI)

## Abstract

Laparoscopic appendectomy is increasingly accepted as the preferred surgical treatment for acute appendicitis and represents one of the most common emergency operations performed in both adult and paediatric populations. However, in patients with perforated appendicitis laparoscopy is associated with an increased incidence of postoperative intraabdominal infections compared to open appendectomy. Nowadays urgent imaging is commonly requested by surgeons when postoperative complications are suspected. Due to the widespread use of laparoscopy, in hospitals with active surgical practices clinicians and radiologists are increasingly faced with suspected postappendectomy complications. This pictorial essay illustrates the normal cross-sectional imaging findings observed shortly after laparoscopic appendectomy and the spectrum of appearances of iatrogenic intraabdominal infections observed in adults and adolescents, aiming to provide radiologists with an increased familiarity with early postoperative imaging. Emphasis is placed on the role of multidetector CT, which according to the most recent World Society of Emergency Surgery (WSES) guidelines is the preferred and most accurate modality to promptly investigate suspected intraabdominal infections and highly helpful for correct therapeutic choice, particularly to identify those occurrences that require in-hospital treatment, drainage or surgical reintervention. In teenagers and young adults MRI represents an attractive alternative modality to detect or exclude iatrogenic abscesses without ionising radiation.

*Teaching points*

• *Laparoscopic appendectomy is the preferred surgical treatment for uncomplicated acute appendicitis*

• *In perforated appendicitis laparoscopy results in increased incidence of intraabdominal infections*

• *Multidetector CT promptly assesses suspected iatrogenic intraabdominal infections*

• *Interpretation of early postoperative CT requires knowledge of normal postsurgical imaging findings*

• *Postsurgical infections include right-sided peritonitis, intraabdominal, pelvic or liver abscesses*

## Introduction

Acute appendicitis (AA) represents one of the most common surgical emergencies worldwide and often occurs in young people, adolescents and children. Nowadays laparoscopic appendectomy (LA) has become increasingly accepted as the preferred surgical procedure in adult patients with suspected or documented AA [1–4].

However, despite several published studies it is still unclear whether the classical open appendectomy (OA) remains superior to LA in terms of efficacy and safety. Although with similar overall short-term morbidity and mortality compared to OA, in adults LA is associated with lower incidence of wound infection, postoperative ileus and analgesics use, an earlier resumption of normal diet, shorter hospitalisation and more rapid recovery to normal activities [3, 5, 6]. Perforated appendicitis, with or without localised or disseminated peritonitis, represents the crucial risk factor for the development of intraabdominal abscesses (IAAs). Infectious complications following laparoscopic treatment of acute uncomplicated (without mural necrosis or perforation) and gangrenous AA occur in 1.1 and 1.6 % of patients respectively, without significant differences compared to open surgery. Conversely, the reported incidence of postoperative IAA in patients with perforated appendicitis is significantly (three to six times) higher with LA (9–24 % of patients) compared to OA (3–4 %) [3, 6–9].

Similarly, in paediatric populations LA currently represents the preferred surgical procedure for most cases of AA and is associated with shorter hospitalisation, earlier resumption of normal diet and activities, and a reduced morbidity including lower incidence of wound infections and postoperative ileus [2, 10]. However, LA remains controversial because of the concern for intraabdominal infections, which occur in 6.4–15 % of all children operated laparoscopically. The reported incidence of IAA significantly differs between patients treated for acute uncomplicated (approximately 2 %) and perforated appendicitis (up to 24–41 %) [7, 10–13]. Conversely, other studies did not report statistically significant differences in the rate of IAA among children following laparoscopic versus open surgery [1, 2].

The reasons underlying this increased rate of postoperative infections are not fully known. Some studies suggested the key role of bacterial contamination at the operative site during aggressive surgical manipulation of the infected appendix, followed by extensive peritoneal irrigation leading to greater serosal contamination. Alternatively, IAA may result from local interstitial infection in the ileocaecal area caused by mesothelial damage from CO_2_ pneumoperitoneum and a local thermal effect from surgical instrumentation [3, 9, 11].

Due to the high number of laparoscopic procedures performed worldwide, in hospitals with active surgical practices clinicians and radiologists may be increasingly faced with suspected postoperative complications following LA. In this article, we review the usual cross-sectional imaging findings observed shortly after appendectomy and the appearances of postoperative abscesses in adolescents and adults, with emphasis on the role of multidetector CT (MDCT) as the preferred and most accurate modality to investigate suspected spontaneous and iatrogenic intraabdominal infections [4].

## Clinical features and indications for postoperative imaging

At our university-based general hospital, emergency department physicians diagnose AA in roughly 140 adult and teenage patients each year, resulting in an average of 65 and 29 LA and OA surgeries respectively. During the last 6 years, at our radiology department 35 patients underwent cross-sectional imaging with MDCT or magnetic resonance imaging (MRI) to investigate suspected postoperative complications following LA, corresponding to approximately one such study every 9 weeks and 1 out of 11 operated patients. According to both literature reports and personal experience, clinical suspicion of postoperative complications most commonly occurs from a few days to a median of 2 weeks after surgery, not unusually (nearly half of our patients) shortly after hospital discharge following a brief, uneventful early postoperative period [7–9].

The most common manifestations of postoperative infections include persisting pelvic, right lower quadrant or diffuse abdominal pain associated with variable degrees of tenderness or frank peritonitis, hypotension, nausea and fever despite treatment with broad-spectrum antibiotics. Elevated leukocyte count and acute phase reactants such as C-reactive protein (CRP) are usually present. Alternatively, in a minority (10–15 %) of patients the clinical presentation is delayed and includes systemic symptoms such as persistent low-grade fever, diarrhoea and weight loss [1, 7–9].

According to our experience, postoperative imaging is mostly indicated when an adult or teenage patient complains of persistent pain within 3 to 6 weeks after LA. Prompt MDCT study is mandatory when physical examination indicates peritonitis or clinical symptoms and laboratory parameters suggest persistent or recurrent systemic inflammation. In our experience we encountered 11 patients with postoperative infectious complications, including 2 cases of localised peritonitis, 7 deep IAAs and 2 patients with liver abscesses. Furthermore, MDCT may occasionally identify conditions unrelated to previous appendicitis, such as caecal diverticulitis or unsuspected Crohn’s ileitis (one case each in our series) [4].

## Imaging techniques and normal postoperative appearances

According to the most recent World Society of Emergency Surgery (WSES) guidelines for the management of intraabdominal infections, MDCT represents the imaging modality of choice for confirmation or exclusion of suspected spontaneous and iatrogenic intraabdominal infections in adult patients. With the current multiplanar high-resolution MDCT imaging, we do not routinely rely on preliminary bowel opacification. However, peroral administration of water-soluble contrast medium at least 45–60 min before scanning may be beneficial in patients with scarce intraabdominal fat planes to differentiate the opacified caecum and distal ileum from abnormal collections. To limit the radiation dose, in most cases we recommend a single MDCT acquisition during the venous phase (75–80 s delay) after intravenous injection of iodinated contrast medium, unless contraindicated by renal function impairment or history of allergy. Additionally, we recommend reconstruction and review of images along axial, coronal and sagittal planes [4].

**Fig. 1:**
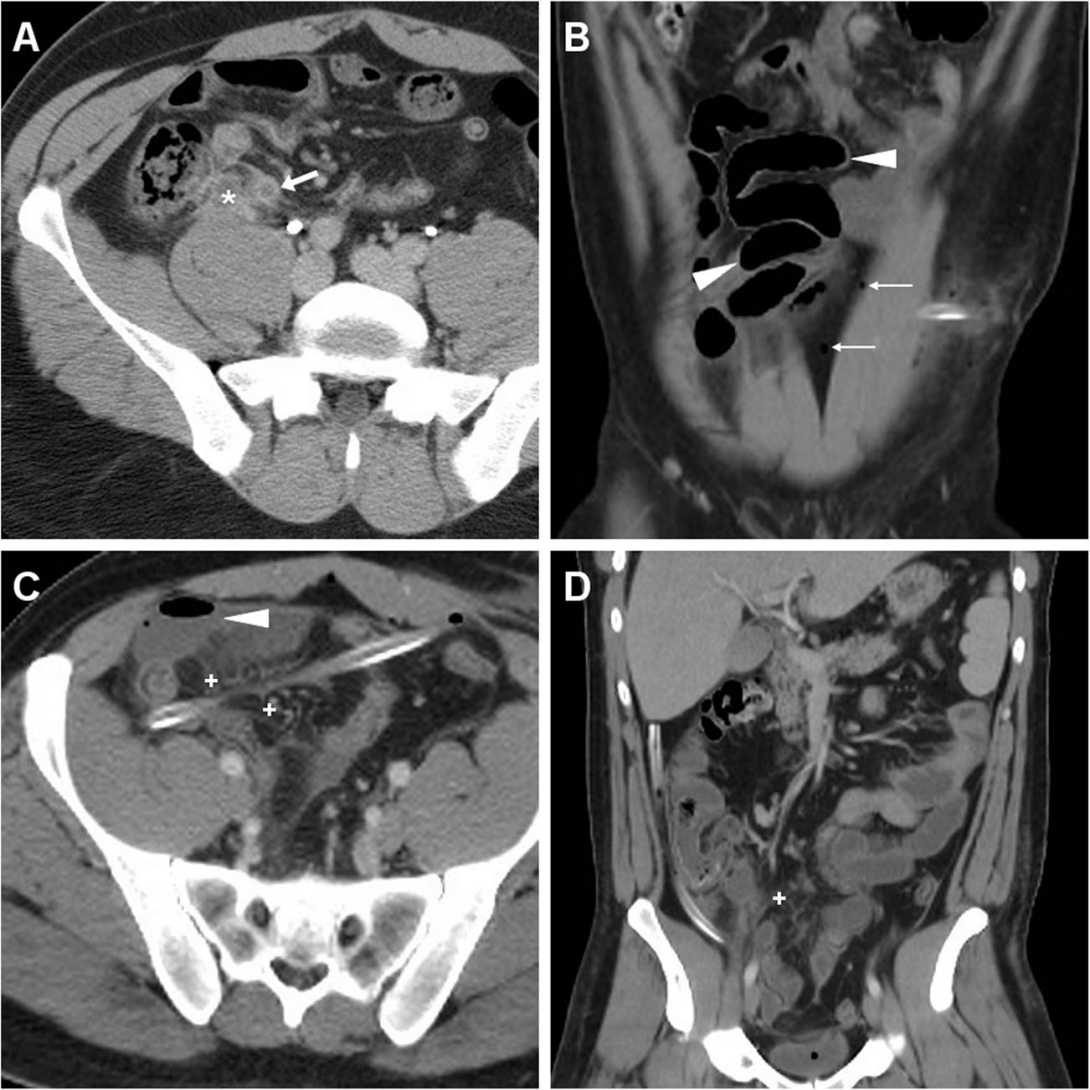
In a 32-year-old male with clinical and laboratory features consistent with acute appendicitis (*AA*), preoperative contrast-enhanced MDCT (**a**) showed a small-sized abscess-like collection (*) abutting the distended appendix (*arrow*) with a moderately enhancing wall and perivisceral inflammatory stranding. Three days after laparoscopic appendectomy (*LA*) a repeated MDCT investigation was performed because of persistent fever, right lower quadrant pain and markedly increased C-reactive protein (384 mg/l). Scattered residual intraperitoneal gas bubbles (*thin arrows* in **b**), distended small bowel loops (*arrowheads* in **b** and **c**) with small air-fluid levels consistent with postoperative ileus, minimal stranding and hypervascularisation (+) of the pericaecal fat planes were seen, without intraabdominal abscesses. Note the drainage tube still in place. The patient recovered fully with intensive antibiotic treatment

**Fig. 2:**
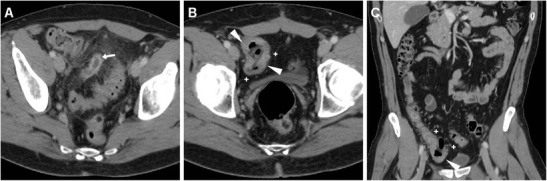
A 51-year-old male with pelvic pain was preoperatively investigated with contrast-enhanced MDCT, which showed classic features of uncomplicated AA (*arrow* in **a**) with periappendiceal inflammatory fat stranding. Three weeks after LA, persistent abdominal pain led to repeated MDCT, which showed normalisation of the pericaecal fat planes (+), absence of ascites and abscess collections, and reactive circumferential thickening (*arrowheads* in **b** and **c**) of the lower caecum. The patient recovered well without any further treatment

In our experience, at least during the first postoperative week after LA, some imaging features such as intraabdominal gas bubbles (Fig. 1), inflammatory-like hyperattenuation (“stranding”) of the pericaecal fat planes adjacent to the site of operation (Fig. 1), minimal peritoneal or mesenterial fluid, distended small bowel loops with air fluid levels consistent with postoperative ileus (Fig. 1) and uniform, circumferential mural thickening of the caecum (Fig. 2) are not associated with IAA and with need for further treatment, and they should therefore not be reported as abnormal. We observed a more or less pronounced oedematous caecal thickening in 7/22 (31.8 %) patients without intraabdominal infections and disorders unrelated to previous surgery, and none of them failed conservative treatment. Furthermore, MDCT is extremely sensitive and represents the gold standard technique for the detection, localisation and quantification of free intraperitoneal air, which is a common but not invariable finding after laparoscopic surgical procedures and may result from residual insufflated gas or from trocar dislodgement during difficult entry or positioning. Since artificial pneumoperitoneum during LA is mostly obtained using carbon dioxide, which is much more rapidly absorbed than room air, intraabdominal gas is usually much smaller and of shorter duration compared to that observed after open surgeries. In our experience, during the first 3–4 days after LA a few nondependent intraperitoneal air bubbles are seen in 25–30 % of patients investigated with MDCT. Conversely, extensive or persisting pneumoperitoneum should suggest the possibility of an anastomotic leak [14, 15].

**Fig. 3:**
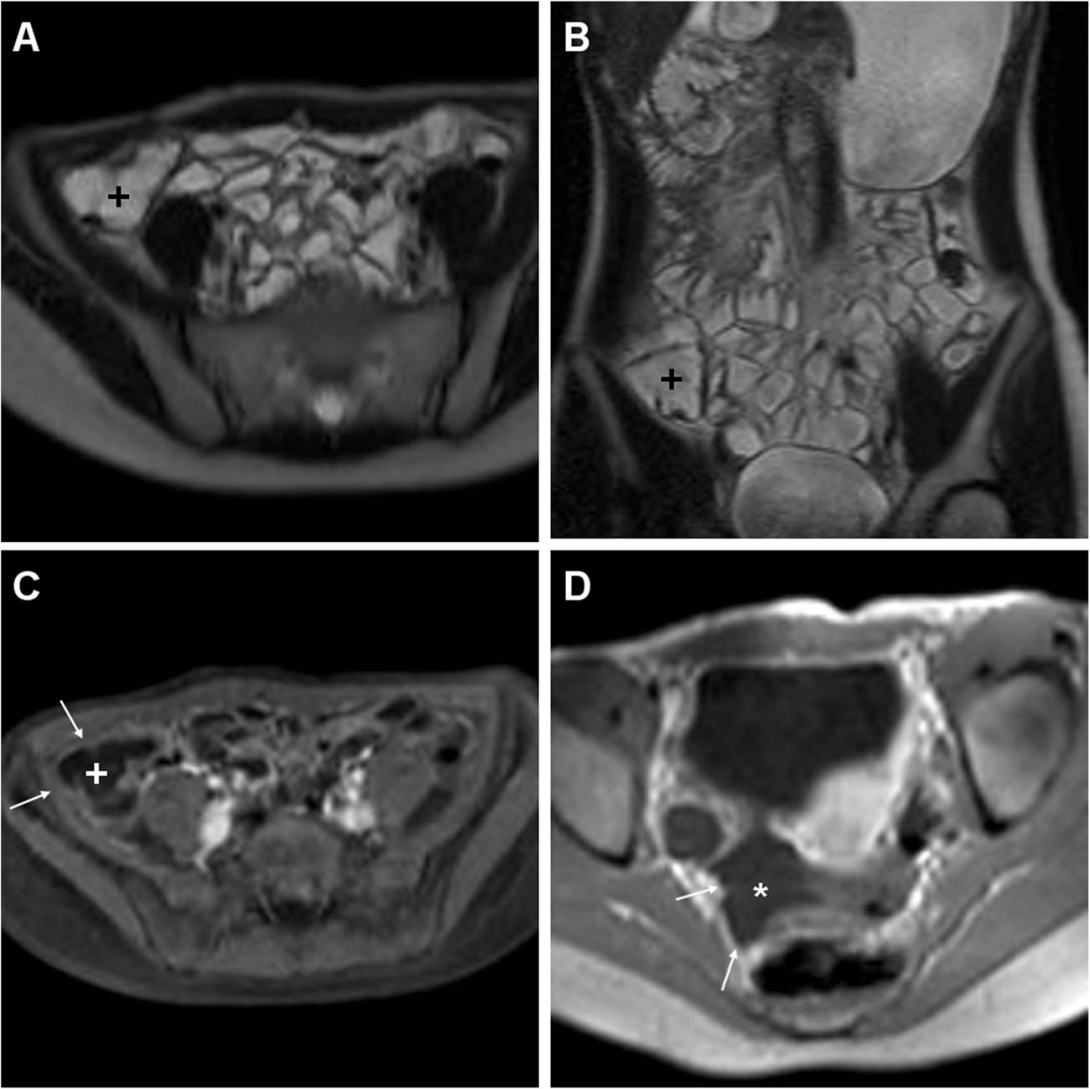
A 16-year-old female with intermittent low-grade fever, failure to thrive and weight loss 15 weeks after LA for uncomplicated AA. Considering the patient’s young age, to avoid use of ionising radiation investigation was carried out by means of MR enterography including peroral bowel distension with diluted polyethylenglycol solution. Axial (**a**) and coronal (**b**) T2-weighted images show optimal ileal and caecal (+) distension by intraluminal fluid, without residual inflammatory fat stranding and appreciable abscess collection. After intravenous gadolinium-based contrast, T1-weighted images with (**c**) and without (**d**) fat suppression exclude abnormal enhancement of the peritoneal serosa (*thin arrows*), with minimal fluid (* in **d**) in the peritoneal cul-de-sac. The patient did not require further treatment

Although with limitations in severely ill or uncooperative patients, due to the concern for use of ionising radiation in teenagers and young adults, MRI is becoming an attractive alternative modality that can prove very helpful to detect or exclude iatrogenic IAA. Current MRI scanners allow robust, time-efficient acquisition protocols including free breathing or respiratory triggering sequences, high-resolution multiplanar T2-weighted images and breath-hold volumetric T1-weighted sequences after intravenous gadolinium contrast. The routine use of T2-weighted fat suppression techniques such as single-shot fast spin-echo or short-tau inversion recovery (STIR) provides better visualisation of inflammatory changes involving the intraabdominal adipose tissue. Furthermore, the integration of diffusion-weighted imaging (DWI) with apparent diffusion coefficient (ADC) measurements in body MRI protocols may prove valuable to detect small IAA and to differentiate inflammatory lesions from ascites [16–18]. Recently, we started using MRI as a first-line alternative to MDCT to investigate cooperative teenagers and young adults. Although our experience with postoperative MRI is still very limited, we observed that inflammatory fat stranding may persist for at least 10 days. Identification of normalised pericaecal tissues without fat stranding (Figs. 1, 2 and 3) at CT or MRI is highly consistent with absence of complications.

## Imaging features of postoperative intraabdominal infections

**Fig. 4:**
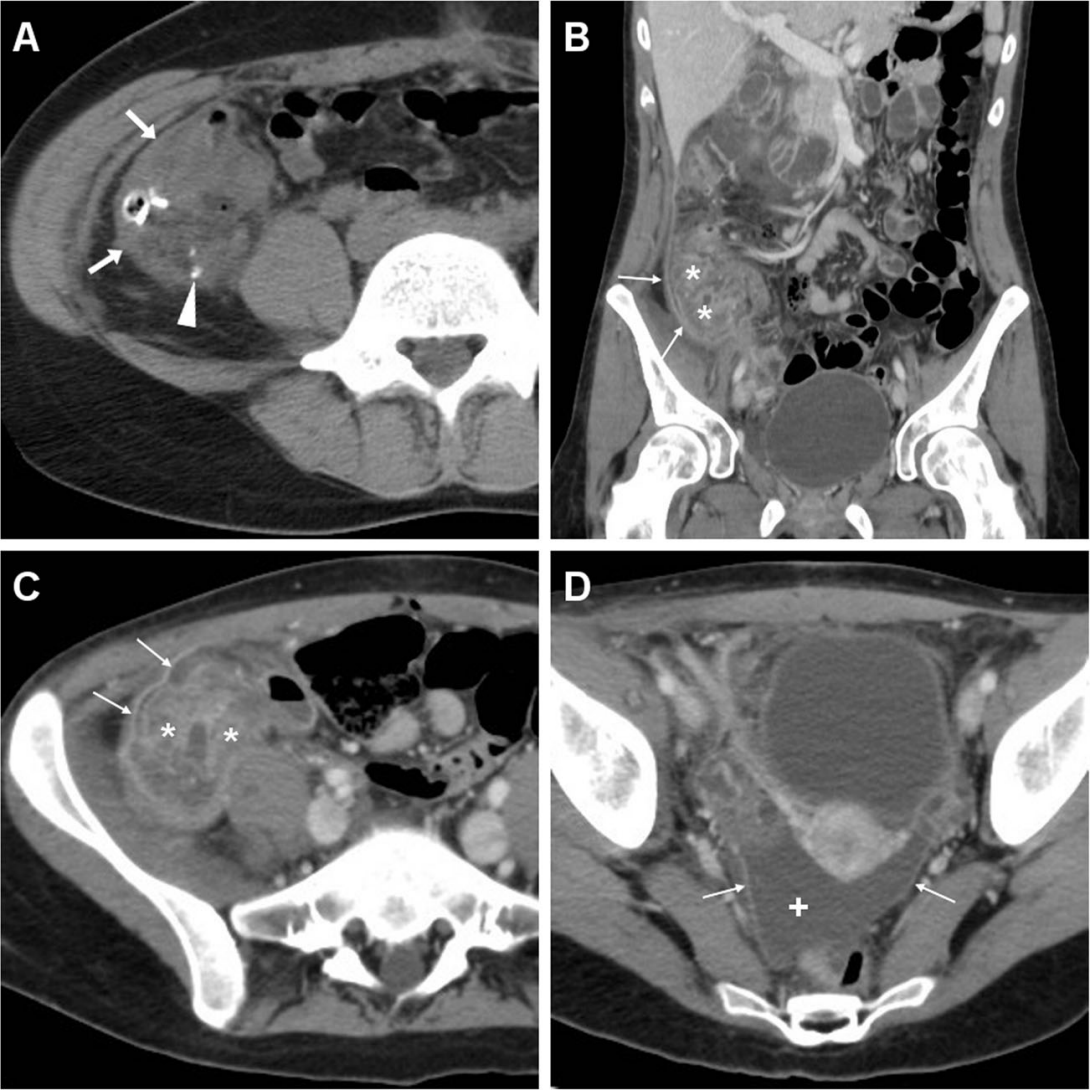
A 22-year-old female with abdominal pain and the clinical finding of peritonitis 3 days after LA for AA. Unenhanced MDCT (**a**) shows thickened peritoneal serosa (*arrows*) abutting the thickened caecum and at least one dependent calcific focus in the right paracolic gutter (*arrowhead*) consistent with a dropped appendicolith. In a different patient, a 26-year-old female with fever, vomiting, leukocytosis (13,000 cells/mmc) and elevated acute-phase reactants (230 mg/l) 9 days after LA for acute uncomplicated AA, multiplanar contrast-enhanced MDCT images (**b**…**d**) reveal hyperenhancing peritoneum (*thin arrows*) in the right parietocolic gutter and iliac fossa (**b**, **c**) abutting the thickened oedematous caecum (*), moderate effusion (+) and thickened serosa in the pelvic cul-de-sac (**d**). Intensive in-hospital antibiotic treatment allowed regression of the clinical and laboratory features, interpreted as postoperative infection without abscess

In the past, the limited radiologic literature dealing with postoperative appearances after appendectomy placed most of the emphasis on the detection of ectopic (“dropped”) appendicoliths expelled from the appendix before or during surgical manipulation as the key sign of failed appendectomy. The hallmark of an appendicolith is a small-sized (4–10-mm) calcification (with attenuation in the range 100 to 1,000 HU) most usually located dependently in the paracaecal region, parietocolic gutter (Fig. 4a) or Morison’s pouch, often within a fluid-like abscess collection [19, 20]. The differential diagnosis of a postoperative intraabdominal hyperdense focus encompasses retained oral contrast material, a surgical clip, dropped gallstone and calcified mesenteric lymph node. When available, careful review of the preoperative MDCT showing features of AA can be helpful. Correlation with a history of biliopancreatic disease and surgery may avoid confusion between dropped gallstones and the rarer appendicoliths, which are both located in the right hemiabdomen in the vast majority of cases [19, 20]. Although dropped appendicoliths can become a source of persistent infection, which may lead to the development of an IAA, in our experience their importance should not be overemphasised, since approximately 30 cases have been reported in the literature, and our patient series includes only one occurrence. Furthermore, MRI unreliably identifies appendicoliths because of the very low signal intensity of calcific bodies [19, 20].

**Fig. 5:**
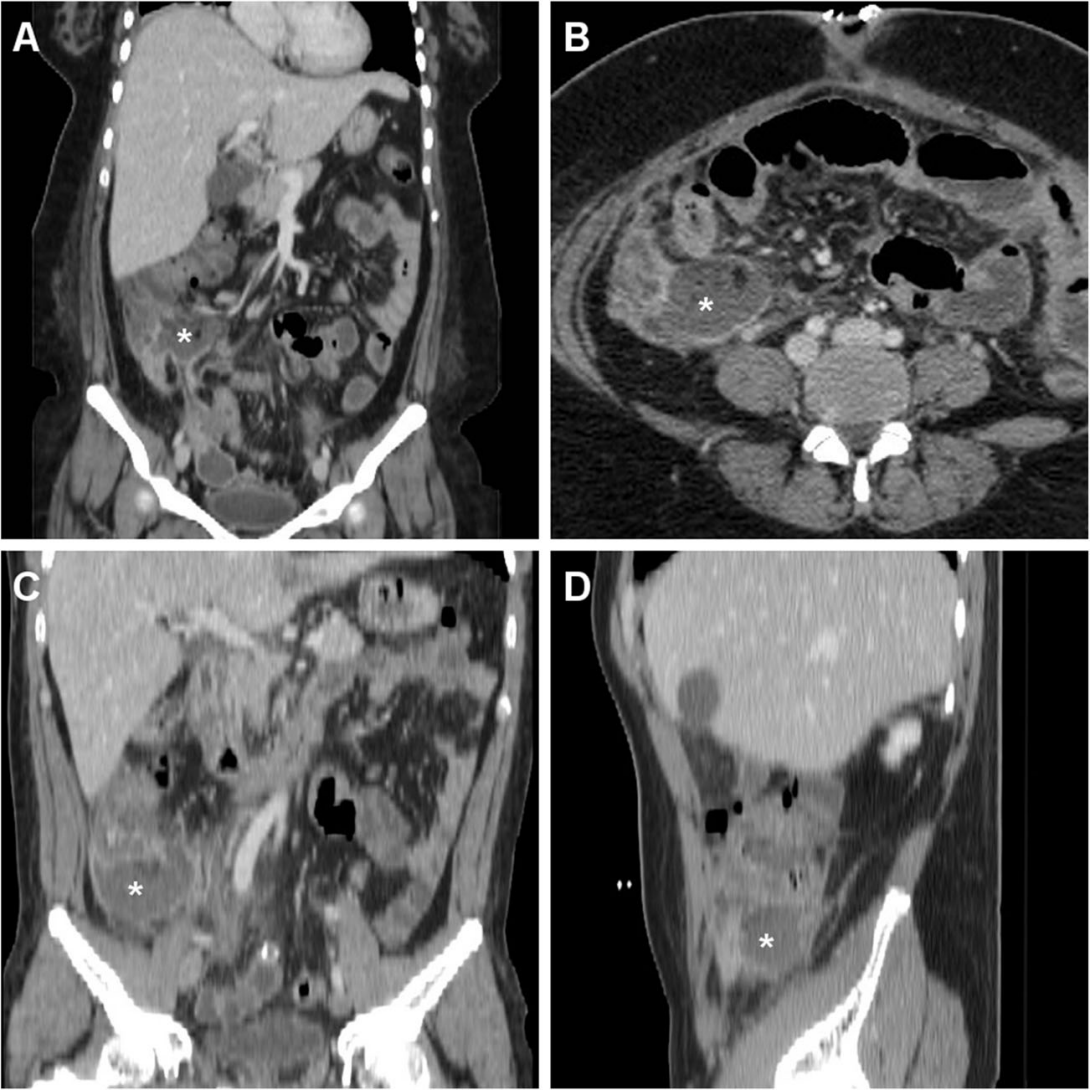
A 38-year-old overweight female with recent LA for gangrenous AA complained of persistent diarrhoea and abdominal pain after hospital discharge, without physical evidence of peritoneal irritation. Laboratory tests showed blood loss and moderate leukocytosis. Coronal (**a**) and axial (**b**) images from contrast-enhanced MDCT revealed a sizeable fluid-like collection (*) abutting the caecum, consistent with the formation of a postoperative abscess, associated with right-sided pleural effusion and atelectasis. Abnormalities regressed at repeated MDCT (not shown) after intensive in-hospital antibiotic treatment. In a different patient, a 36-year-old male with right lower quadrant pain and persistent elevated C-reactive protein (130 mg/l) and leukocytosis 6 days after LA for abscessualised AA, multiplanar images from contrast-enhanced MDCT (**c**, **d**) showed a large abscess (*) with fluid-like contents and enhancing walls that occupies the infracaecal region. Percutaneous drainage obtained regression of the purulent collection

**Fig. 6:**
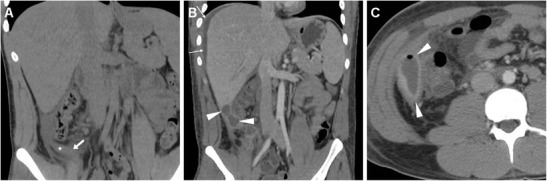
A 22-year-old male with AA preoperatively diagnosed with unenhanced MDCT (**a**) revealing a distended, thickened appendix (*arrow*) with a small calcified appendicolith, surrounded by right-sided fascial fluid and perivisceral fat stranding. Intraoperative and pathological findings diagnosed phlegmonous AA. Two weeks after LA, persistent fever, leukocytosis (13,500/mmc) and increased lipase and C-reactive protein values (380 mg/l) led to repeated contrast-enhanced MDCT. Imaging showed subphrenic (*thin arrows* in **b**) and infrahepatic (*arrowheads* in **b** and **c**) collections: the former with enhancing serosa, the latter with thickened enhancing borders abutting the lateroconal fascia. Laparoscopic exploration confirmed subhepatic and right subphrenic abscesses; conversion to laparotomy was necessary to drain collections and clean the peritoneal cavity. The subsequent postoperative course was further complicated by right-sided pneumonia and pleural effusion

**Fig. 7:**
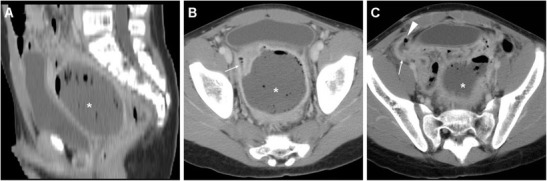
In a 17-year-old male with clinical evidence of peritonitis 12 days after LA for AA at another hospital, contrast-enhanced MDCT showed the pouch of Douglas occupied by a vast abscess (*) with thickened enhancing borders and fluid content with gas bubbles, which compressed the urinary bladder anteriorly and communicated (*thin arrows*) with another smaller collection in the right iliac fossa (*arrowheads*). Laparotomy confirmed pelvic and pericaecal abscesses, and the surgical procedure necessitated positioning of a temporary ileostomy

In our experience, iatrogenic intraabdominal infections after LA may sometimes (2/11 cases, 18 %) manifest at MDCT as localised peritonitis without IAA, including moderate effusion, slightly thickened and hyperenhancing peritoneal serosa in the right parietocolic gutter, iliac fossa and pelvic cul-de-sac (Fig. 4b–d). Conversely, most postoperative infectious complications are represented by variable-sized abscess collections, showing characteristic MDCT features such as fluid-like content and commonly gas bubbles, surrounded by a thickened, often irregularly enhancing wall. In the largest published series, the majority of IAAs were located in the right lower quadrant or pelvis in 61 and 22.5 % of patients respectively [8]. Accordingly, we observed postoperative deep abscesses most commonly (four cases out of seven occurrences) in the pericaecal region (Fig. 5), sometimes in the right parietocolic gutter and/or ipsilateral subphrenic space (Fig. 6), or occupying the pelvic inlet or Douglas’ pouch (Fig. 7). Mild, uniformly enhancing peritoneal thickening may be associated (Fig. 6). Right-sided pleural effusion and basal lung atelectasis-pneumonic consolidations are present in nearly half of these patients [8].

At MRI, IAAs appear as centrally nonenhancing fluid-like collections with layering low signal intensity debris, a thick gadolinium-enhancing peripheral rim, and bright signal on diffusion-weighted images indicating restricted diffusion [16–18].

## Miscellaneous complications

**Fig. 8:**
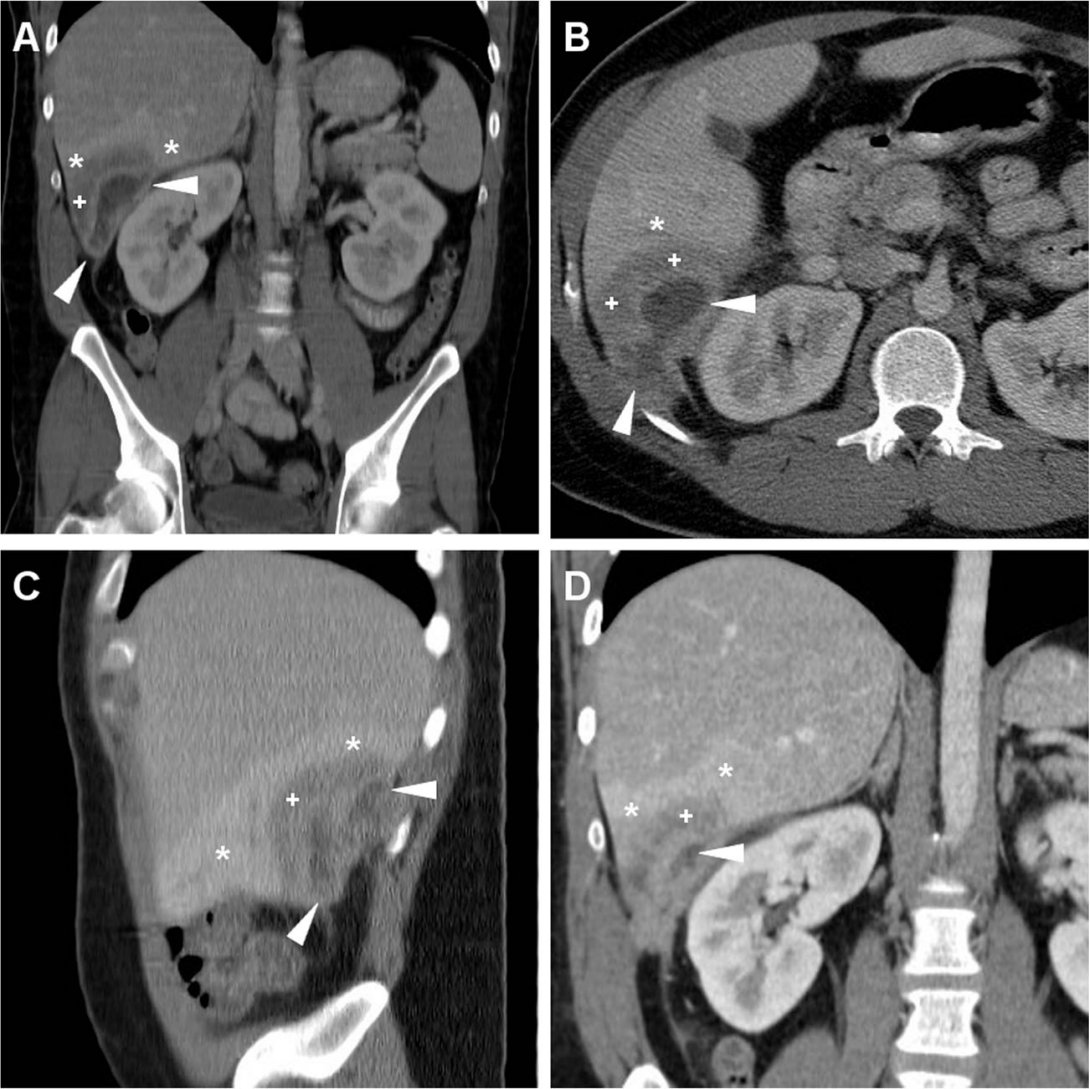
A 24-year-old male presented with mild fever, right lower quadrant pain and absent peritoneal irritation for a week, 3 months after LA for AA. Following ultrasound detection of a poorly defined hypoechoic lesion in the right liver lobe, multiplanar images from contrast-enhanced MDCT (**a**–**c**) showed a bilobated abscess in the sixth liver segment, with a strongly enhancing rim (*arrowheads*) and hypodense centre, surrounded by hypoattenuating oedema (+) and hyperenhancing liver parenchyma (*). Percutaneous drainage was not feasible because of the limited size of liquefied content at ultrasound. After antibiotic treatment, the lesion decreased in size at repeated MDCT (**d**) and ultimately resolved

Very rarely postoperative infections after LA may give rise to single or multifocal pyogenic liver abscesses indistinguishable from those caused by intestinal and biliary infections. Usually heralded by persistent fever, pain and leukocytosis despite broad-spectrum antibiotics, iatrogenic liver infections are associated with a high mortality and need intensive antibiotic treatment, percutaneous or surgical drainage. In two patients investigated at our hospital, MDCT revealed single or multiple (one case each) hepatic abscesses with an enhancing periphery and hypoattenuating centre, variably surrounded by oedematous and/or hyperenhancing liver parenchyma (Fig. 8) [21, 22].

Exceptionally, intraabdominal infections may seed via a patent processus vaginalis leading to the formation of a scrotal abscess [23].

Furthermore, as with other abdominal surgeries, the portal venous system should be carefully scrutinised for signs of dilatation and filling defects. Following appendectomy pylephlebitis has been occasionally reported to involve the superior mesenteric vein, particularly in patients with underlying coagulopathy or when the operation is performed in advanced stages of the disease with peritonitis [24].

## Conclusion

Appendectomy remains one of the most common emergency surgical procedures and is increasingly performed using laparoscopy. Partly due to fear of litigation, urgent diagnostic imaging is increasingly requested by surgeons when postoperative complications are suspected after laparoscopic procedures. Prompt cross-sectional imaging investigation with MDCT and familiarity with the normal postoperative findings and the imaging appearances of IAA are helpful for accurate diagnosis and correct therapeutic choice, particularly to identify those occurrences that require prolonged in-hospital treatment, drainage or surgical reintervention. When available, MRI represents a valuable alternative modality that allows avoiding ionising radiation in young patients and adolescents in good clinical condition [4, 15].
